# Human Papillomavirus Genotype Distribution in Invasive Cervical Cancer in Pakistan

**DOI:** 10.3390/cancers8080072

**Published:** 2016-07-30

**Authors:** Asif Loya, Beatriz Serrano, Farah Rasheed, Sara Tous, Mariam Hassan, Omar Clavero, Muhammad Raza, Silvia De Sanjosé, F. Xavier Bosch, Laia Alemany

**Affiliations:** 1 Department of Pathology, Shaukat Khanum Memorial Cancer Hospital and Research Centre Lahore, Punjab 54000, Pakistan; asifloya@skm.org.pk (A.L.); drrazamuhammadi@gmail.com (M.R.); 2 Unit of Infections and Cancer (UNIC), Cancer Epidemiology Research Programme, Catalan Institute of Oncology (ICO)–IDIBELL, L’Hospitalet de Llobregat, Gran Via de l’Hospitalet, 199-203, 08908 L’Hospitalet de Llobregat, Barcelona, Spain; bscarro@iconcologia.net (B.S.); stous@iconcologia.net (S.T.); oclavero@iconcologia.net (O.C.); x.bosch@iconcologia.net (F.X.B.); 3 Clinical Research Office, Shaukat Khanum Memorial Cancer Hospital and Research Centre Lahore, Punjab 54000, Pakistan; crc3@skm.org.pk (F.R.); crc@skm.org.pk (M.H.); 4 Unit of Infections and Cancer (UNIC), Cancer Epidemiology Research Programme, Catalan Institute of Oncology (ICO)–IDIBELL, L’Hospitalet de Llobregat, Gran Via de l’Hospitalet, 199-203, 08908 L’Hospitalet de Llobregat, Barcelona, Spain; CIBER Epidemiología y Salud Pública (CIBERESP). Madrid, Spain; s.sanjose@iconcologia.net

**Keywords:** human papillomavirus, cervical cancer, genotype, epidemiology, human papillomavirus vaccines, Pakistan

## Abstract

Few studies have assessed the burden of human papillomavirus (HPV) infection in Pakistan. We aim to provide specific information on HPV-type distribution in invasive cervical cancer (ICC) in the country. A total of 280 formalin-fixed paraffin-embedded tissue blocks were consecutively selected from Shaukat Khanum Memorial Cancer Hospital and Research Centre (Lahore, Pakistan). HPV-DNA was detected by SPF10 broad-spectrum PCR followed by DNA enzyme immunoassay and genotyping by LiPA25. HPV-DNA prevalence was 87.5% (95%CI: 83.0–91.1), with 96.1% of cases histologically classified as squamous cell carcinoma. Most of the HPV-DNA positive cases presented single infections (95.9%). HPV16 was the most common type followed by HPV18 and 45. Among HPV-DNA positive, a significantly higher contribution of HPV16/18 was detected in Pakistan (78.4%; 72.7–83.3), compared to Asia (71.6%; 69.9–73.4) and worldwide (70.8%; 69.9–71.8) and a lower contribution of HPVs31/33/45/52/58 (11.1%; 7.9–15.7 vs. 19.8%; 18.3–21.3 and 18.5%; 17.7–19.3). HPV18 or HPV45 positive ICC cases were significantly younger than cases infected by HPV16 (mean age: 43.3, 44.4, 50.5 years, respectively). A routine cervical cancer screening and HPV vaccination program does not yet exist in Pakistan; however, the country could benefit from national integrated efforts for cervical cancer prevention and control. Calculated estimations based on our results show that current HPV vaccine could potentially prevent new ICC cases.

## 1. Introduction

Invasive cervical cancer (ICC) is the fourth most common cancer and the fourth leading cause of cancer related deaths amongst women worldwide, with nearly 85% of the global burden occurring in developing countries [1].

Human papillomavirus (HPV) infection is recognized as one of the major causes of infection-related cancers worldwide and represents an important global public health problem [2]. HPV infection is a sexually transmitted infection commonly found in the anogenital tract of men and women with and without clinical lesions, with peak prevalence in young sexually active individuals. The vast majority (70%–90%) of HPV infections is asymptomatic and resolve spontaneously in 1–2 years [3]. Persistent HPV infection is a well-established cause of ICC with HPV virtually causing 100% of squamous cell carcinoma (SCC) cases and more than 85% of adenocarcinoma (ADC) cases [3,4,5]. The vast majority of HPV-related cancers are ICC cases (more than 85%), but HPV is also aetiologically linked with variable fractions of other anogenital cancers (anus, vulva, vagina and penis) and head and neck cancers (particularly the oropharynx), and it is also the causal factor in genital warts and recurrent respiratory papillomatosis [2]. More than 200 different HPV subtypes exist; more than 40 infect the genital tract, and of these 12 are classified as carcinogenic [3,4,5]. Data confirm HPVs16/18/45/33/31/52/58/35, in decreasing rank order, as the eight most frequently detected types in ICC, contributing to more than 90% of ICC globally [6,7,8].

Pakistan is currently the 6th most populous country in the world with 92 million women in 2015. This figure is expected to increase to up to 151 million by 2050 [9]. The magnitude of cervical cancer is difficult to estimate in the country because national cancer registries are lacking and the ones that exist only cover certain urban areas. Cervical cancer remains the 3rd leading cause of female cancer, and the 3rd cause of female cancer deaths in Pakistan. In 2012, there were an estimated 5233 new cases and 2876 ICC deaths, with incidence and mortality rates of 5.9 and 3.2 per 100,000; that are expected to rise. ICC is also the 2nd most common cancer and the 5th leading cause of cancer deaths in women aged 15 to 44 years [1].

The role of HPV infection in cervical cancer in the Pakistani population has not been broadly studied. Few studies have been done, and they provide little evidence, due to the small sample size, lack of consistency in results, and patchy representation of the country [10,11,12,13,14]. In addition, these studies have focused largely on Southern regions and specifically in the city of Karachi. Only a recent study has explored HPV infection in the North of Pakistan, where most of the population is living. The rates of high risk (HR) HPV infection obtained in these studies are provided in Table 1.

Being a lower-middle income country with a high burden of cancer, Pakistan lacks an effective nationwide screening and HPV vaccination program for ICC and most women are diagnosed of cancer at a late stage, when the survival rates are low. In Pakistan, there is only limited opportunistic screening, which is mostly based on visual inspection using acetic acid (VIA) [11,15,16]. Countries with resource constraints, like Pakistan, often lack personnel with technical expertise and public health infrastructure to provide Papanicolaou smear and cervical cytology [17]. HPV testing is another cervical cancer screening option that provides a 30%–40% gain in sensitivity for detecting cervical intraepithelial neoplasia (CIN) grade 2+ compared to cytology at the cost of a 3%–5% lost in specificity, and allows safe extension of the intervals between screening episodes [18,19]. In addition, HPV testing technologies are exploring simpler and more accessible sampling devises and testing methods (such as “self-sampling”) that can facilitate and increase participation in screening programs while reducing the cost and complexity.

HPV prophylactic vaccines using virus like particles (VLP) have been recognized as an effective intervention to control cervical cancer and other HPV-related cancers in both men and women [20]. Recent recommendations from WHO suggest implementation of a coordinated and wide ranging strategy to prevent all HPV related disease including cervical cancer. Such programmes should target public education to increase awareness of risk factors for acquiring HPV infections, training of medical personnel on HPV screening techniques and improving information and access for screening, diagnosis and treatment of cervical cancer. Vaccination should be used as a primary prevention tool and should not undermine focus of national strategies for developing effective screening programmes since the HPV vaccine does not protect against all HPV types [20]. Currently available bivalent HPV vaccines—CervarixTM (2vHPV) (GlaxoSmithKline Biologicals, Rixensart, Belgium)—and quadrivalent HPV vaccine—Gardasil^®^ (4vHPV) (Merck & Co., Inc., Kenilworth, NJ, USA)—prevent HPVs16/18 infections, which account for almost 70% of ICC worldwide [7,8]. A novel broad spectrum 9-valent HPV vaccine—Gardasil^®^9 (9vHPV) conferring protection against five other common HPV types (HPVs31/33/45/52/58) has been recently approved [21,22]. All HPV vaccines also prevent other HPV-related cancers. WHO recommends the administration of HPV vaccines, if possible, before the onset of sexual activity, i.e., before first exposure to HPV infection [20]. Other potential primary prevention tools to avoid HPV infection include increased condom use, delay of age at first intercourse, and reduction in the number of sexual partners. These interventions have proven to be effective in reducing sexually transmitted infections and human immunodeficiency virus transmission, although their specific efficacy in terms of reducing cervical cancer incidence remains unknown [23].

Information on HPV genotype distributions is crucial for planning cervical cancer interventions. As mentioned, limited data is available in Pakistan, making it essential that further research be conducted on the burden of HPV-related cancers, HPV prevalence and type distribution in the general population of the country. This information would help to develop a comprehensive intervention policy, taking into account the religious and sociocultural aspects of the society for future management of cervical cancer [24]. Thus, the present study is aimed at providing baseline information about HPV prevalence and type distribution in the Pakistani population with ICC. This information is crucial for stakeholders to formulate the appropriate strategies toward cervical cancer prevention and control in the country.

## 2. Results

### 2.1. General Characteristics

**Figure 1:**
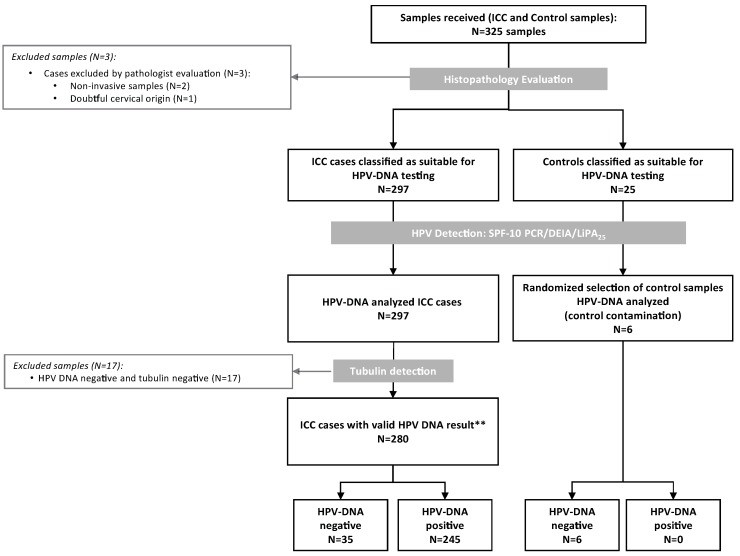
Algorithm of the inclusion criteria of the Pakistan invasive cervical cancer cases in the study. HPV: Human Papillomavirus; ICC: Invasive Cervical Cancer; ** Valid cases: those that tested HPV-DNA positive or HPV-DNA negative with a positive tubulin result.

Three-hundred-twenty-five formalin-fixed paraffin-embedded tissue blocks were consecutively selected from Shaukat Khanum Memorial Cancer Hospital and Research Centre (SKMCH & RC), Lahore, Pakistan between 2005 and 2010. Histological evaluation confirmed 300 samples as cervical specimens, but three of them were dismissed because two were non-invasive tumours and one of the conventional SCC was of doubtful cervical origin. The remaining 25 samples were controls. The 297 ICC cases classified as suitable for HPV-DNA testing and a random sample of six controls was HPV-DNA analyzed (Figure 1). We excluded 17 samples negative for both HPV-DNA and tubulin. Consequently, the final analysis included 280 ICC samples with valid HPV-DNA results.

The mean age of the ICC cases at the time of diagnosis was 49.5 years (standard deviation-sd. 12.4). The majority of histological diagnoses were SCC (96.1%; 95%CI: 93.1–98.0), with two ADC and nine cases classified as other histological diagnoses. Invasion was higher than 50% in 65.7% (59.8–71.3) of the cases, and the presence of necrosis was less than 50% in 98.2% (95.9–99.4) of the cases. Ten cases (3.6%) had a pre-neoplastic cervical intraepithelial neoplasia (CIN) grade 3 adjacent to the neoplastic lesion (Table 2).

### 2.2. HPV Prevalence and Type Distribution

HPV-DNA prevalence was 87.5% (83.0–91.1) (Table 2). Among HPV-DNA positive ICC, most of the cases presented as single infections (95.9%) and only two cases harbored more than one HPV type (0.8%) (Table 3). In eight cases, HPV-DNA was identified by DNA Enzyme Immunoassay (DEIA), but LiPA25 was negative and were finally classified as HPV undetermined (HPVX). Type specific distribution among HPV positive cases is described in Table 2. HPV16 was the most detected type, with 67.3% (61.1–73.2) relative contribution (RC) as single infection. HPV18 and HPV45 were the following most frequently detected types, with RCs of 10.2% (6.7–14.7) and 7.3% (4.4–11.4), respectively. No low risk (LR) HPV types were identified.

**Figure 2:**
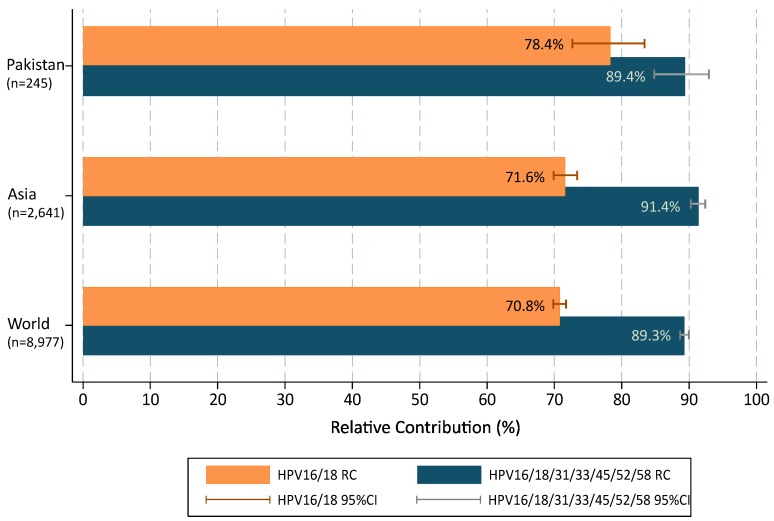
HPV type relative contribution in invasive cervical cancer cases from Pakistan positive for HPV, compared to Asia and Worldwide. 95%CI: 95% confidence interval. Additional information: multiple infections are computed according to a proportional weighting attribution [25,26].

When multiple infections were added to single types in accordance with a proportional weighting attribution, the overall contribution of HR types target by 2vHPV and 4vHPV vaccines (HPVs16/18) were 78.4% (72.7–83.3); increasing up to 89.4% (84.8–92.9) with the five additional types (HPVs31/33/45/52/58) included in the 9vHPV vaccine (Figure 2). Compared to data from broader geographical areas extracted from De Sanjose et al. [7], the contribution of HPVs16/18 was higher in Pakistan than in the Asian region or worldwide (single and multiple infections combined were 78.4% vs. 71.6% and 70.8%, respectively; *p* = 0.029) but lower for HPV31/33/45/52/58 (11.1% vs. 19.7% and 18.5%, respectively; *p* = 0.003). The RC increased up to 89.4%, 91.4% and 89.3%, when combining the seven HR HPV types included in the 9vHPV vaccine and the differences among Pakistan, Asia and worldwide became minimal and not statistically significant.

**Figure 3:**
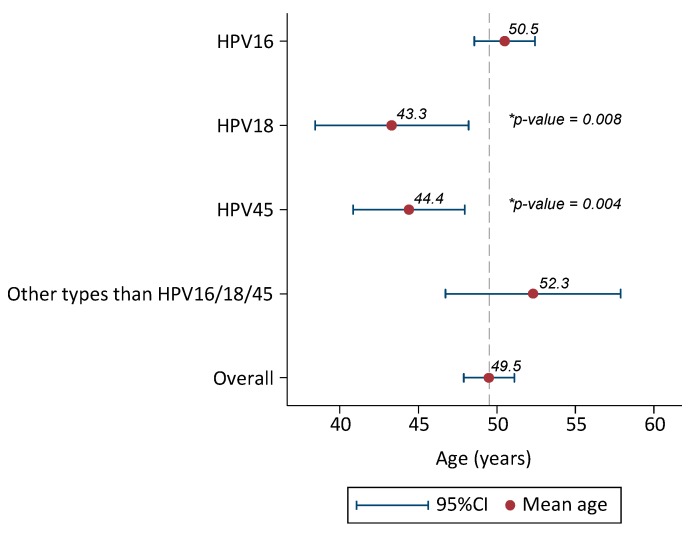
Mean age and 95%CI of HPV-positive invasive cervical cancer cases from Pakistan (only single HPV infections considered). 95% CI: 95% confidence interval. Additional information: Comparison of mean age by HPV types (HPV16 reference category) with ANOVA test. *p*-Value statistically significant for HPV18 and HPV45.

Mean age of cases infected with HPV16 was 50.5 years old (48.6–52.4). Cases infected with HPV18 were 7 years younger than HPV16, mean age 43.3 (38.4–48.2) (ANOVA test *p*-value = 0.008). Similarly, HPV45 positive cases were six years younger than HPV16, mean age 44.4 (40.8–47.9) (*p* = 0.004). Mean age of cases infected with HPV types different to HPV16/18/45 was 52.3 (46.7–57.9) (Figure 3) (Supplementary Materials: Table S1).

Without changes in prevention and control in Pakistan, and only due to population growth, projected global estimates of ICC cases attributable to HPV16/18 are expected to rise from 4250 in 2015 to 6998 new cases in 2050 and from 596 to 982 new cases attributable to HPV 31/33/45/52/58.

## 3. Discussion

Data on type specific HPV infection in normal women and in women with cervical lesions is essential for assessing the potential impact of HPV vaccination and HPV testing for cervical cancer screening. The role of HPV infection in ICC is limited in Pakistan. To the best of our knowledge, this is the largest study reporting specifically on HPV prevalence and genotype distribution in women with ICC in Pakistan. The cases belong to an international study using standardized protocols and centralized HPV testing technology [7].

HPV prevalence in evaluable cases was 87.5% in our study. Data on overall HPV prevalence in ICC from Pakistan shows some variability, ranging from 18% to 98% [10,11,12,13,14] (Table 1). Since it is generally accepted that HPV virtually causes 100% of SCC ICC cases, the contrasting results could be partly explained by differences in the sensitivity of the HPV detection techniques used, the different histologies of the cases included in each study and the quality of the biological specimen, among others. In the present study, ICC cases HPV DNA negative were subject to polymerase chain reaction (PCR) targeting the human tubulin to evaluate the quality of DNA. Seventeen samples were both negative for HPV DNA and tubulin and therefore excluded for the final analyses.

Our data supported HPV16 as the most common type in Pakistan (67.3% as single infection among HPV-DNA positive ICC cases), followed by HPV18 (10.2%). These results are in agreement with the international data [6,7], but differences are noted when comparing with figures from local studies [10,11,12,13,14] (Table 1). This can be a consequence due to differences in techniques used for HPV detection, but also to the study setting and the specific characteristics of the population included (age, histology of the samples included…etc.).

The third most common detected type in our study was HPV45, as it was already observed worldwide and in most world regions, including Western/Central Asia. Regional variation in the ranking of other HPV subtypes (HPV33, HPV52, HPV58) has been already noted in Asia, with higher contribution of HPV52 and 58 in Eastern Asia compared to Western/Central Asia countries [6,7,27].

HPV16 cases were older compared to HPV18 or HPV45 ones, mean age: 50.5, 43.3 and 44.4 years, respectively. Our results are in accordance with world estimates and suggest a rapid progression to ICC of HPV18/45 [7,28].

Multiple HPV infections were very low (0.8%), with one case co-infected with HPV16/18 and other with HPV16/45. While some studies from Pakistan show similar low rates of co-infection a recent study conducted in Punjab registered higher rates of HPV16/18 co-infection (34.0%) [11,12]. The use of different primers for HPV-DNA detection can explain the differences in the sensitivity to detect HPV infections.

Conventional squamous cell carcinoma was the commonest (96.1%) histological type. The histopathological distribution is similar to previous studies from Pakistan [11,29].

The present study strengthens the knowledge of HPV prevalence and type distribution in cervical cancer cases in Pakistan. Although current incidence rates of ICC in Pakistan (5.9 per 100,000) are lower than world ones, ICC remains the third leading cause of female malignancies in the country. Pakistan is a more conservative culture, resulting in a lower prevalence of HPV and other sexual transmitted infections [16]. However, current changes in sexual behavior including increasing pre-marital sexual activity can lead to changes in HPV infection, and ICC rates are expected to increase [1,30]. A National Health program for cervical cancer screening, prevention and control is needed. For Pakistan, a lower middle income country, low cost options for screening and awareness of cervical cancer etiology can be used effectively to cover the masses. Nevertheless, HPV vaccines can play a vital role despite economic and cultural issues. Cost-effectiveness analyses in developing countries indicate that HPV vaccinations supplemented with screening may be a cost-effective strategy to reduce incidence and mortality by cervical cancer. However, concerns about affordability still remain for low income countries [31]. Pakistan is qualified for GAVI support (Global Alliance for Vaccines and Immunization), and could access the vaccine at a much better price. Demonstration programmes launched in several countries suggest that GAVI supported HPV programmes are successfully demonstrating the feasibility of vaccinating adolescent girls with reported 60%–90% coverage rates [32]. Hence, both the affordability and value for money of HPV vaccine will need to be favourable, as it will compete with money being spent on other existing national immunization initiatives [31].

The findings observed in the present study reveal that currently licensed 2vHPV and 4vHPV vaccines may potentially prevent close to 78% of the ICC cases in Pakistan. We have also tried to estimate the burden of cases prevented by cross protection of the 2vHPV and 4vHPV vaccines for Pakistan, Asia and the world. Cross protection efficacy data have been obtained from FUTURE I/II and PATRICIA clinical trials, as these studies had much the same length of follow-up [33,34]. We have used the information provided by the trials about the efficacy of the 2vHPV and 4vHPV vaccine against CIN2+ due to non HPV vaccine types and by including lesions co-infected with HPV16 or HPV18, in women who were HPV-naive at baseline. When taking into account the potential cross-protection conferred by the 2vHPV and 4vHPV vaccines against types not targeted by those vaccines, up to 9.9% and 5.1% cases could be additionally prevented by the 2vHPV and 4vHPV vaccines, respectively [33,34]. Comparatively, up to 14.4% and 8.0% cases could be additionally prevented by cross protection of 2vHPV and 4vHPV vaccines in Asia and up to 14.5% and 8.6% worldwide, respectively (Table 4). Hence, these data should be taken with caution as the inclusion of lesions co-infected with HPV16/18 may overestimate the vaccine efficacy against lesions caused by non-vaccine types because co-infected lesions are more common in control groups than vaccine groups [33,34,35]. Finally, the addition of HPVs 31/33/45/52/58 in the 9vHPV vaccine could potentially prevent almost 90% of ICC lesions in Pakistan, similar to worldwide data [7] (Table 4).

## 4. Materials and Methods

### 4.1. Study Design and Materials

The present study is part of an international retrospective cross-sectional study to estimate the HPV DNA detection and genotype distribution in ICC lesions worldwide. The project was designed and coordinated by the Catalan Institute of Oncology (ICO), Barcelona, Spain and the DDL Diagnostic laboratory in Rijswijk, The Netherlands. An anonymized collection of formalin-fixed paraffin-embedded tissue blocks were subsequently selected from Shaukat Khanum Memorial Cancer Hospital and Research Centre, Lahore, Pakistan. In August 2013, 300 cervical cancer specimens were received. In addition, the center sent 25 paraffin blocks to be used as controls (containing non HPV-related specimens). Information about year of diagnosis, age at the time of diagnosis and original pathological diagnosis was also provided. Shaukat Khanum Memorial Cancer Hospital and Research Centre (SKMCH & RC) is a tertiary cancer hospital and charitable institute. It has established itself as a center of excellence providing comprehensive care free to cancer patients irrespective of their ability to pay (about 75% patients get financial support); however, patients registered with us belong to diverse range of socio-economic strata and geographic areas. Nearly 74.6% belonged to the Province of Punjab (north of Pakistan) where 60% of the total population of the country lives, and SKMCH & RC is situated in its provincial capital [36]. The ICC cases included in this study are a mix of patients getting treatment at SKMCH & RC and patients consulting for diagnostic purposes. All protocols were approved by the clinical research ethics committee of SKMCH & RC.

### 4.2. Pathology and Laboratory Procedures: Formalin Fixed Paraffin Embedded Blocks Processing, Histopathological Evaluation, HPV DNA Detection and Typing

Paraffin blocks were processed under strict conditions. Pathology and laboratory procedures were performed at ICO as previously described [7]. Briefly, re-embedding of the tissue material was done if necessary when the paraffin block was in poor conditions for cutting. Microtome sectioning of the specimens under sterile conditions and sandwich technique were carried out to confirm an optimal number of sections to be used for pathology evaluation, DNA extraction, HPV-DNA detection, and HPV genotyping. At least four paraffin sections were obtained for each block (sandwich method). The first and last sections were stained with Hematoxylin and Eosin (H&E) for histopathological evaluation and in between sections were used for HPV DNA testing. Histopathological evaluation included confirmation of ICC, histological type (squamous cell carcinoma, adenocarcinoma, adenosquamous carcinoma, other types), presence of pre-neoplastic lesions adjacent to ICC, degree of necrosis and of tumor infiltration (quantity of necrosis/infiltrating component of the tumor in the total of the section), and adequacy of the sample to proceed for HPV testing. A sample was determined adequate for HPV testing if ICC was observed in both the first and last stained sections. Cases difficult to classify, cases with a discordant diagnosis compared to the original diagnosis and all the rare histological types were further reviewed by two senior expert pathologists at ICO. Intermediate sections were used for HPV DNA detection and genotyping. DNA detection was performed by PCR with SPF-10 broad spectrum primers. The amplified PCR products were tested for the presence of HPV DNA using a DNA enzyme immunoassay (DEIA) that recognized at least 54 mucosal HPV genotypes. Borderline samples were run on the DEIA system again. Tubulin was used to evaluate the quality of DNA. All HPV DNA-negative samples in the study were subjected to a PCR targeting the human tubulin gene (forward primer: TCCTCCACTGGTACACAGGC; reverse primer: CATGTTGCTCTCAGCCTCGG), which generated a 65-bp amplicon, the same size as the SPF-10 amplicon used for assessing the presence of HPV DNA [37]. Samples that were both negative for HPV DNA and tubulin were excluded for the final analyses. Amplimers testing positive for viral DNA by DEIA were genotyped with a reverse hybridization line probe assay—LiPA25 that detects 25 HR and LR types 6/11/16/18/31/33/34/35/39/40/42/43/44/45/51/52/53/54/56/58/59/66/68/70/74). The sequence variation within the SPF-10 interprimer region allows the recognition of these different HPV genotypes, but not types 68 and 73 because their interprimer regions are identical and cannot be distinguished with this test.

Control paraffin blocks were tissues unrelated to HPV that were processed at the same time as the cases. These controls were used to determine contamination during the inclusion process at the laboratory of origin.

### 4.3. Statistical Analysis

Variables included in the analysis were age at diagnosis, year of diagnosis, histopathological diagnosis, presence of non-neoplasic epithelium, presence of preneoplastic lesions (grade if any), presence of necrosis and tumor infiltration (as percentage of total), and HPV DNA detection and typing.

To compare HPV DNA positivity among variables, we used Pearson’s chi-squared test or Fisher exact test when necessary. ANOVA test was performed to compare mean age among HPV DNA positive and negative. Evaluations of trends by age, year at diagnosis, percentage of necrosis and percentage of invasion were determined by trend test analysis for proportions. Statistically significant *p*-value was set at 0.05.

HPV type-specific RC of the cases was also estimated. RC refers to the percentage of positive samples for a specific HPV type in relation to all the HPV-positive samples. HPV-type specific information for Pakistan only included single infections because only two specimens harbored multiple infections.

RC of HPV types included in current vaccines in Pakistan were compared to Asia and the world, using data from De Sanjose et al. [7]. For these comparisons, the contribution of multiple infections was also assessed. In cases with multiple infections, more than one specific HPV type was detected in the cervical lesion. However, it should be noted that each lesion is produced by one HPV type alone. For the present study, multiple infections were added to single types in accordance with a proportional weighting attribution where single HPV types were used as references. For example, if two cases with ICC lesions were found to test positive for both HPV 16 and 45, and there were nine cases infected by HPV-16 as a single type and one case infected by HPV 45 as a single type, then [2 × 9/(9 + 1)] or 1.8 of these two multi-type infected lesions would be attributed to HPV 16 and [2 × 1/(9 + 1)] or 0.2 attributed to HPV 45 [25,26].

Estimation of new ICC cases potentially preventable by current HPV vaccines were calculated for the year 2015 and projected to year 2050, for Pakistan. Estimations were based on the following assumptions and data sources: (a) HPV virus is necessary for the development of ICC, so we assumed that the attributional fraction (AF) for each HPV type in ICC corresponded to the RC in CC; (b) Globocan 2012 incidence rates were used with the assumption that these rates will apply in the future. Globocan incidence rates were estimated as the weighted average of the local rates from South Karachi (1998–2002), Punjab, Lahore district (2008–2010) and Quetta (1998–1999) [1]; (c) the population forecast available in the latest World Population Prospects (revision 2015) was used [9]; and (d) impact of vaccination was not taken into account. Model-based predictions of long-term vaccine benefits are dependent on several uncertain assumptions and hence such weighted attributions should be interpreted with caution.

## 5. Conclusions

This study strengthens the knowledge of HPV prevalence and type distribution in ICC in Pakistan and highlights the importance of national level programmes for cervical cancer screening and control in the country. A high contribution of HR HPV types 16, 18 and 45 in cervical cancer cases in Pakistan is consistent with worldwide and regional data. Current HPV vaccines could potentially prevent 78.3% of ICC cases related to HPVs16/18 in Pakistan, increasing to almost 90% with the inclusion of HPVs31/33/45/52/58. Our data can help policy makers and public health professionals formulate the most appropriate strategies for cervical cancer prevention and control in the country. Further research is needed to collect data on screening and prevention strategies being used in the country and their potential impact on cervical cancer control.

## Figures and Tables

**Table 1:** Studies assessing human papillomavirus prevalence and type distribution in invasive cervical cancer in Pakistan.

Reference	Period of Study	Cities	Age (Years)	PCR/Primers	Tested (N)	Prevalence (%, 95CI)	Multiple Infections (%)	HPV Tested (RC%)	Details on Histology (N)
Gul et al., 2015 [10]	2010–2013	Islamabad, Rawalpindi	21–80	GP5/GP6; TS16; TS18; Beta globin	56	91.1 (80.4–97.0)	Not specified	HPV16 (45.1%). HPV18 (43.1%). other types (11.8%)	SCC (33), ADC (23)
Siddiqa et al., 2014 [11]	2007–2010	Punjab	25–70	GP5+/GP6+; TS16; TS18; C16E7; C18E7, Beta globin	47	97.9 (88.7–99.9)	34.0	HPV16 (32.6%). HPV18 (28.3%). HPV1618 (34.8%). other types (4.3%)	SCC (43), ADC(1); ADSC (3)
Raza et al., 2010 [12]	2004–2008	Karachi	15–59	GP5+/6+; Beta globin	91	91.2 (83.4–96.1)	3.3	HPV16 (83.1%). HPV18 (7.2%). HPV33 (1.2%). HPV42 (1.2%). HPV45 (4.8%). HPV56 (2.4%). HPV59 (1.2%). HPV66 (1.2%). HPV69 (1.2%)	SCC (79), ADC (3), Small cell (4), Other (5)
Yousuf et al., 2010 [13]	2003–2008	Karachi	25–90	My09/My11; GP5+/6+	50	18.0 (8.6–31.4)	Not specified	HPV16 (55.6%). unknown (44.4%)	SCC (50)
Khan et al., 2007 [14]	1991–2005	Karachi	20–60	GP5/GP6, TS16; TS18; Beta globin	60	98.3 (91.1–100)	Not specified	HPV16 (94.9%). HPV18 (1.7%). other types (3.4%)	SCC, ADC (N not specified)

HPV: Human papillomavirus; PCR: Polymerase Chain Reaction; N: Number; RC: Relative Contribution; SCC: squamous cell carcinoma; ADC: Adenocarcinoma; ADSC: Adenosquamous carcinoma. Additional information: all studies used paraffin-embedded tissue samples [10,11,12,13,14].

**Table 2:** Characteristics of the invasive cervical cancer cases from Pakistan and HPV-DNA prevalence.

	ICC Cases		HPV-DNA Prevalence		Chi-Squared/Fisher’s Exact Test
				
**Characteristics**	**N**	**% ^a^**	**N+**	**% ^b^**	***p*-value**
***Age at diagnosis***					
**Mean (Sd.)**	49.5 (12.4)		HPV-DNA +: 49.5 (12.5)		
***Year of diagnosis***					
**2005**	44	15.7	38	86.4	
**2006**	51	18.2	41	80.4	
**2007**	53	18.9	46	86.8	
**2008**	47	16.8	44	93.6	
**2009**	26	9.3	24	92.3	0.500
**2010**	59	21.1	52	88.1	0.246#
***Histological evaluation***					
**Squamous cell carcinoma**	269	96.1	236	87.7	
**Adenocarcinoma**	2	0.7	1	50.0	
**Other diagnosis:**	9	3.2	8	88.9	0.304
**Undifferentiated carcinoma**	4	44.4	3	75.0	
**Neuroendocrine carcinoma**	3	33.3	3	100.0	
**Lymphoepithelioma-like carcinoma**	1	11.1	1	100.0	
**Trophoblastic carcinoma**	1	11.1	1	100.0	
***% invasion***					
**≤50%**	96	34.3	84	87.5	
**>50%**	184	65.7	161	87.5	1.000
***% necrosis***					
**≤50%**	275	98.2	242	88.0	
**>50%**	5	1.8	3	60.0	0.119
***Presence of pre-neoplastic lesions adjacent to the neoplastic lesion***					
**No**	270	96.4	235	87.0	
**CIN3**	10	3.6	10	100.0	0.619
**Total**	**280**	**100.0**	**245**	**87.5**	

ICC: Invasive cervical cancer; Sd.: Standard deviation; HPV: Human papillomavirus; N: Number of ICC cases; N+: number of ICC cases positives for HPV-DNA; CIN: cervical intraepithelial neoplasia; #: trend test *p*-value. ^a^ Column percentages: distribution of the cases for each variable. ^b^ Row percentages: HPV positivity for each variable.

**Table 3:** Human papillomavirus type distribution among HPV-DNA positive cases of invasive cervical cancer from Pakistan.

HPV Type	ICC Cases	
	N-Positive	RC (%)
***Single HPV infections***	***235***	***95.9***
**HPV16**	165	67.3
**HPV18**	25	10.2
**HPV45**	18	7.3
**HPV56**	5	2.0
**HPV68or73**	5	2.0
**HPV31**	3	1.2
**HPV52**	3	1.2
**HPV33**	2	0.8
**HPV35**	2	0.8
**HPV39**	2	0.8
**HPV59**	2	0.8
**HPV66**	2	0.7
**HPV58**	1	0.4
***Multiple HPV infections***	***2***	***0.8***
**HPV16 & HPV18**	1	0.4
**HPV16 & HPV45**	1	0.4
***HPVX***	***8***	***3.3***
**Total**	**245**	**100.0**

ICC: Invasive cervical cancer; N-positive: Number of ICC cases HPV positive; RC: relative contribution; HPV: Human papillomavirus; HPVX: Undetermined type.

**Table 4:** Potential impact of licensed human papillomavirus vaccines to prevent invasive cervical cancer cases in Pakistan, compared to Asia and Worldwide.

HPV Types of ICC Cases Potentially Preventable through Vaccination	Pakistan			Asia			World		
	(N = 300; N-pos = 245)			(N = 2994; N-pos = 2641) ^a^			(N = 10,575; N-pos = 8977) ^a^		
	N-pos	RC (%)	95%CI	N-pos	RC (%)	95%CI	N-pos	RC (%)	95%CI
**Cases infected with HPV types targeted by prophylactic vaccines (HPV16/18)**	192	78.4	72.7–83.3	1892	71.6	69.9–73.4	6357	70.8	69.9–71.8
**Cases infected with HPV types non-targeted by prophylactic vaccines potentially preventable by cross-protection conferred by 4vHPV vaccine (HPV31/33/35/39/45/51/52/56/58/59) (including co-infection HPV16/18) ^b^**	12	5.1	2.6–8.4	212	8	7.0–9.1	736	8.2	7.6–8.8
**Cases infected with HPV types non-targeted by prophylactic vaccines potentially preventable by cross-protection conferred by 2vHPV vaccine (HPV31/33/35/39/45/51/52/56/58/59/68) (including co-infection HPV16/18) ^c^**	24	9.9	6.4–14.2	381	14.4	13.1–15.8	1306	14.5	13.8–15.3
**Cases infected with HPV types targeted by prophylactic 9vHPV vaccine (HPV6/11/16/18/31/33/45/52/58)**	219	89.4	84.8–92.9	2416	91.5	90.4–92.5	8032	89.5	88.8–90.1

HPV: Human papillomavirus; ICC: Invasive cervical cancer; *N*: Number of ICC cases; N-positive: Number of ICC cases HPV positive; RC: Relative contribution; 95% CI: 95% confidence interval. Additional information: Inclusion of HPV 16 and 18 co-infected lesions can overestimate cross protection efficacy of the vaccine against lesions with nonvaccine type HPVs, because co-infected lesions will be more common in control groups than vaccine groups. **^a^** Data obtained from De Sanjose et al. [7]. **^b^** Cross-protection conferred by 4vHPV vaccine for composites HPV31/33/35/39/45/51/52/56/58/59 (including co-infection HPV16/18) is estimated at 32.5% (95%CI: 6.0–51.9) [33]. **^c^** Cross-protection conferred by 2vHPV vaccine for composites HPV31/33/35/39/45/51/52/56/58/59/68 (including co-infection HPV16/18) is estimated at 56.2% (95%CI: 37.2–65.0) [34].

## Supplementary Materials

Supplementary File 1

## Abbreviations

The following abbreviations are used in this manuscript:

ADC	Adenocarcinoma
ADSC	Adenosquamous carcinoma
AF	Attributional Fraction
CIN	Cervical Intraepithelial Neoplasia
DEIA	DNA Enzyme Immunoassay
H&E	Hematoxylin and Eosin
HPV	Human Papillomavirus
HR	High Risk
ICC	Invasive Cervical Cancer
ICO	Institut Català d’Oncologia (Catalan Institute of Oncology)
LR	Low Risk
N	Number
PCR	Polymerase Chain Reaction
RC	Relative Contribution
SKMCH & RC	Shaukat Khanum Memorial Cancer Hospital and Research Centre
SCC	Squamous Cell Carcinoma
Sd	Standard Deviation
VIA	Visual Inspection using Acetic acid
VLP	Virus Like Particles
2vHPV	bivalent HPV vaccine
4vHPV	quadrivalent HPV vaccine
9vHPV	ninevalent HPV vaccine
95% CI	95% Confidence Interval
