# Supplementary material for: Human Papillomavirus Genotype Distribution in Invasive Cervical Cancer in Pakistan
Source: Cancers (Basel). 2016 Jul 30;8(8):72. doi: 10.3390/cancers8080072 (PMC4999781; doi:10.3390/cancers8080072)
Supplement: Supplementary File 1 [file cancers-08-00072-s001.docx]

Supplementary Materials: Human Papillomavirus Genotype Distribution in Invasive Cervical Cancer in Pakistan

Asif Loya, Beatriz Serrano, Farah Rasheed, Sara Tous, Mariam Hassan, Omar Clavero, Muhammad Raza, Silvia de Sanjosé, F. Xavier Bosch and Laia Alemany

**Table S1.** Mean age of HPV-positive invasive cervical cancer cases from Pakistan, by HPV types (only single infections considered).

|  | **ICC Cases** | | |
| --- | --- | --- | --- |
| **HPV Type (Single Infections)** | ***N*** | **Mean Age (Years)** | **95%IC** |
| ***Single HPV infection*** | ***235*** | ***49.5*** | ***(47.9–51.1)*** |
| **HPV16** | 165 | 50.5 | (48.6–52.4) |
| **HPV18** | 25 | 43.3 | (38.4–48.2) |
| **HPV45** | 18 | 44.4 | (40.8–47.9) |
| **HPV56** | 5 | 60.2 | (45.9–74.5) |
| **HPV68or73** | 5 | 43.6 | (31.4–55.8) |
| **HPV31** | 3 | 58.0 * | (42–65) |
| **HPV52** | 3 | 55.0 * | (42–80) |
| **HPV33** | 2 | 60.5 * | (56–65) |
| **HPV35** | 2 | 31.5 * | (23–40) |
| **HPV39** | 2 | 47.5 * | (30–65) |
| **HPV59** | 2 | 62.5 * | (60–65) |
| **HPV66** | 2 | 48.5 * | (37–60) |
| **HPV58** | 1 | 50.0 * | NA |
| ***Combinations of types*** |  |  |  |
| **HPV16/18** | 190 | 49.5 | (47.7–51.4) |
| **Other HPV typesnot 16/18** | 45 | 49.1 | (45.4–52.8) |
| **HPV16/18/45** | 208 | 49.1 | (47.4–50.8) |
| **Other HPV typesnot 16/18/45** | 27 | 52.3 | (46.7–57.9) |
| **Alpha 7 (18/45/39/59)** | 47 | 44.7 | (41.5–48.0) |
| **Alpha 9 (16/31/33/35/52/58)** | 176 | 50.6 | (48.7–52.5) |

ICC: Invasive cervical cancer; HPV: Human papillomavirus; N-positive: Number of ICC cases HPV positive; 95%CI: 95% confidence interval.
